# Effect of Whole Body Massage by Patient's Companion on the Level of Blood Cortisol in Coronary Patients

**DOI:** 10.5812/nms.13781

**Published:** 2013-09-15

**Authors:** Mohsen Adib-Hajbaghery, Rahman Rajabi-Beheshtabad, Ali Abasi

**Affiliations:** 1 Trauma Nursing Research Center, Kashan University of Medical Sciences, Kashan, IR Iran; 2 Department of Nursing, Dehdasht Imam Khomeini Hospital, Yasouj University of Medical Sciences, Yasouj, IR Iran; 3 Department of Cardiology, Shahid Beheshti Hospital, Kashan University of Medical Sciences, Kashan, IR Iran

**Keywords:** Massage therapy, Relatives, Coronary patients, Cortisol

## Abstract

**Background:**

Inconsistent results have been reported on the effect of massage therapy on the blood cortisol levels. Also, no study is available about the effect of massage done by patient's companions on the level of blood cortisol in patients hospitalized at CCU.

**Objective:**

The present study aimed to evaluate the effect of whole body massage performed by patient's companion on the level of blood cortisol among the patients admitted in CCU.

**Patients and Methods:**

A randomized controlled trial was conducted on 60 patients admitted to a CCU ward. Patients were randomly placed into two groups of massage performed by patient's companion and the control group. In the intervention group, whole body massage was administered. The control group did not receive massage. Data analysis was performed using the SPSS 11.5 software. Independent sample and Paired samples t-test, Chi Square and Fisher's Exact tests were used to analyze the data.

**Results:**

The mean age for the patients was 58.90 ± 15.63 years. None of them had the history of massage therapy. In the group massaged by the patients' companions, the mean of blood cortisol was 323.6 ± 162.6 nanomoles which decreased to 268.4 ± 141.1 after the intervention (P < 0.102). The mean of blood cortisol in the control group did not change significantly.

**Conclusions:**

Massage therapy lowered the level of cortisol in the group massaged by the patients' companions. It can be recommended that massage therapy be used in patients admitted in CCU.

## 1. Background

Acute coronary syndrome and chronic myocardial infarction are among the commonest reasons for hospitalization at coronary care units (CCU) (1). Affliction to these diseases is often accompanied by great fear and anxiety over the likelihood of death (2) and the feeling of anxiety remains with patients and their families for some time (3). Some studies have shown that hospitalization at technologic wards such as CCU is also accompanied with anxiety (4). Factors such as lack of familiarity with environmental conditions, being far from family members, alarms sounds and other environmental and psychological stimulants increase the level of anxiety among patients at CCU wards (5). One study has indicated that 50% of patients experience signs of anxiety following acute coronary syndrome and myocardial infarction (6). Another study in Iran has reported that 90% of these patients experience signs and symptoms of anxiety (7).

Anxiety leads to palpitation, shivering, chest pain and its aggravation, feeling choked and dyspnea, fear of losing control and fear of death (8). This anxiety increases the oxygen demand of heart and accordingly increases the risk of cardiac dysfunction, dysrhythmia, ischemia, inefficiency and the likelihood of patient's death (9).

Some studies have shown that anxiety and stress could lead to the activation of the hypothalamus-hypophysis-adrenal axis and an increase in the secretion of blood cortisol (10-12). There are different methods for lowering patients' stress and anxiety; however, medication treatments are extensively used for lowering stress in patients hospitalized at CCU ward (13).

Considering the side effects of medical treatments, alternative methods may be used rather than drug therapy to decrease anxiety in patients (14). One of the supplementary therapies recommended for lowering anxiety is massage therapy (14-17). Some researchers have reported that lowering stress and anxiety reduces the level of blood cortisol (18). Lindgren et al., have reported that massage therapy leads to a reduction in salivary cortisol (11). Field et al. also examined the effect of massage therapy on the level of cortisol and reported that this method reduces the level of cortisol up to 31% (19). In another study, Field et al., reported that psychotherapy alongside massage therapy for 30 minutes bring about a reduction in the level of cortisol (20). Moyer et al., have also reported that massage therapy might increase the activity of the Vagus nerve and decrease stress-related hormones (18). Nonetheless, Billhult et al., and McVicar et al., did not find a significant difference in the level of salivary cortisol level between massage therapy group and control group (21, 22).

## 2. Objective

Regarding the existing controversies about the effect of massage therapy on the level of cortisol and the fact that no study is available about the effect of massage therapy by patient companions on the level of blood cortisol in the patients hospitalized at CCU, and also because of high workloads of nurses and this issue that patients might be more comfortable to receive massage by their relatives, and relatives also might feel more active in patients care, the present study was conducted with the aim of evaluating the effect of massage therapy performed by patients` companion on the level of blood cortisol among patients admitted in CCU wards.

## 3. Patients and Methods

A randomized controlled trial was conducted on 60 patients admitted to the CCU wards of the Kashan university of Medical sciences. The eligible subjects for this research were identified through daily referring to the CCU wards of Shahid Beheshti Hospital, Kashan, Iran, and going over their hospitalization records alongside consulting with the treating physician.

The inclusion criteria included of being male, hospitalization at CCU ward, having the medical diagnosis of acute coronary syndrome or myocardial infarction, being literate, being completely conscious, having a three-day record of hospitalization, willingness to participate in the study. Also not having the following conditions were selected as additional inclusion criteria : a history of cardiac arrest during the recent 72 hours, being under treatment by warfarin, any coagulation disorder, a known psychological disorder, a cardiac pacemaker, a history of second degree burn in more than 25% of the body surface, a known infectious disease or hepatitis and jaundice, a known adrenal gland disorder, a known skin problem, fever, limb amputation, bone fracture in recent two months, deep vein thrombosis, a dialysis fistula in limbs, and a history of massage therapy. Exclusion criteria included of a reduction in the level of consciousness, bradycardia (less than 60 beats per minute), severe or exertional dyspnea and any hemodynamic instability.

**Figure 1.**
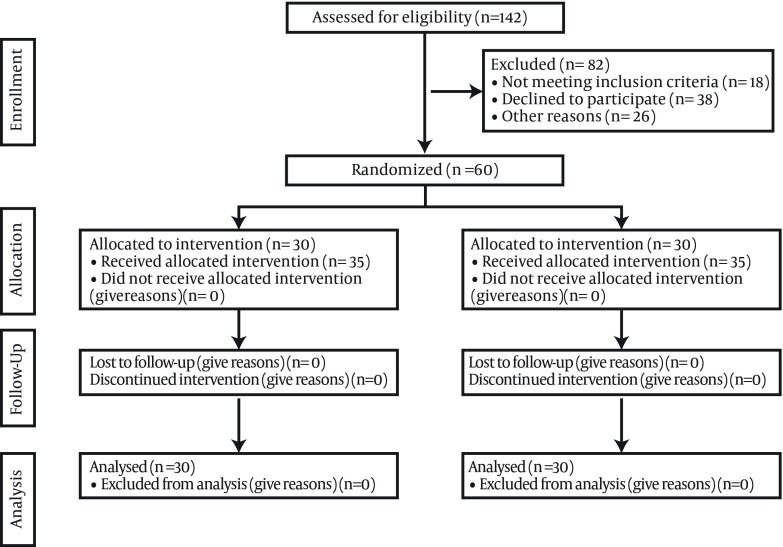
The Study Follow Diagram

Sample size was calculated based on a pilot study on seven samples in which the mean of blood cortisol was 333.89 and decreased to 278.77 after the massage therapy. The difference in the response was 55.12 ± 93.25. Then 24 pairs of subjects were estimated to be needed (and we selected 30 pairs) to be able to reject the null hypothesis that this response difference is zero with a power of 0.8. The Type I error probability associated with this test was 0.05. Sampling was performed via the convenience sampling method and the patients were randomly assigned into control and experiment groups, so that the sample size was completed for each group. The process of sampling was shown in the Figure 1. 

The data collection instrument consisted of two parts. The first part included a demographic data form (age, marital status, occupation, number of children, patient's level of education, and patient's companion level of education). Also, the medical diagnosis as inscribed in the file, and the history of hospitalization were extracted from the patient’s file. The second part of the instrument consisted of one form for recording the amount of cortisol both before and after getting massage.

In the intervention group, massage therapy was administered in a private setting (in a private room or after pulling curtains around the patient) in the third day of hospitalization (due to going through the acute phases of the disease and training the patient's companion for performing massage) after getting permission from the treating physician and obtaining the patient's consent. After the selection of patients and filling in the consent form in the intervention group, the patients’ demographic and clinical data were recorded in a special form and also their blood samples were analyzed for the level of cortisol and the results were recorded in the form.

For each individual in the intervention group, one 60-minute session of massage therapy was performed by one of his relatives. One of the male companions or relatives of patient was selected by consulting with the patient and was trained the manner of performing massage and accordingly evaluated. In case of being accepted, he was allowed to give massage to his patient. Training each of the companions was performed on an individual basis in a 2-hour session in the practice room at the nursing department on a human manikin by the second researcher who had a specialized degree in the field of massage therapy.

Almond oil was used for facilitating whole body massage in back, buttocks, shoulders, deltoid muscles, arms, forearms, palm of both hands and fingers, the posterior part of thighs and ankles, foot soles, the front part of thighs and back of feet and toes, patient's abdomen and chest, axillaries and neck muscles. The techniques used in massage therapy included static massage, superficial stretching technique, stretching massage, lymph vacuuming technique, latitudinal rubbing technique, and myofacial releasing technique (23), which were accompanied with effleurage (gentle and light press while rubbing oil on body) (24). Massage was not performed in painful areas and areas with burn, bruise, inflammation, thrombosis, ecchymosis, and skin wounds.

Half an hour before and 15 minutes after the termination of massage therapy (while patient was relax at least for 15 minutes), the blood sample was obtained for measuring the level of cortisol. To this aim, four milliliters of blood was taken from the patient's arm in a lab tube which was then placed in an ice box and transferred to lab. All patients were massaged in the evening shift and their blood samples were accordingly taken. The level of blood cortisol was identified by using the Immunotech Kits (Beckman Coulter Co. Czech) and according to the instructions given by the manufacturing company and using Gama Counter sets, model Genesys Gamma-1TM, made by LTI Company (Laboratory Technologies INE) in America. In addition, patients were monitored during the massage therapy session.

No intervention was performed for the control group and the patients in this group merely received the routine care of the ward. Blood sample was taken in this group for identifying the level of blood cortisol after filling in the informed consent form and collecting the patients' demographic and clinical data in two rounds with a 105 minutes interval.

### 3. 1. Ethical Considerations

This study was approved by the Research Council and the Research Ethics Committee of Kashan University of Medical Sciences, Kashan, Iran. All the participants in this study signed the informed consent form and were assured of the confidentiality of their individual information and of the lack of coercion for taking part in the study. Data collection was performed after making coordination with the head-nurse at ward and the treating doctor. The research objective was explained to all the study subjects.

### 3. 2. Data Analysis

After entering the data into the SPSS 11.5 software, the descriptive and analytical tests were used. Independent sample t test was used to compare the mean of cortisol in the two groups. Paired samples t-test was used for comparing the pre and post-intervention cortisol in each group, and the Chi Square test and the Fisher's Exact test were used to analyze the nominal and categorized data. In all tests, the level of significance was considered to be 0.05.

## 4. Results

The mean of age for the research subjects was 58.90 ± 15.63 years. Among them, 90% were married and 10% were single or widowed. Also, 65% of patients currently had a job and 35% were either jobless or had been retired. None of them had the history of massage therapy. Regarding education, 70% were at elementary level and 30% were at the high school or over. Most of the selected companions (76.7%) were the patients’ sons and the others were their brothers or friends. Most of the companions (86.7%) were at high school education level. In total, 95% of patients in the intervention group were very much or highly satisfied with massage therapy (Table 1). 

The mean of blood cortisol level before the intervention was 323.6 ± 162.6 nanomoles in the intervention group which was decreased to 268.4 ± 141.1 after the intervention (P < 0.102). The mean of blood cortisol level in the control group both before and after massage did not change significantly (Table 2). 

**Table 1.** The Patients’ Characteristics

	Intervention, No. (%)	Control, No. (%)	P value
**Age, Mean ± SD**	61.1 ± 13.6	56.6 ± 17.3	0.27
**Medical diagnosis**			0.08
Acute coronary syndrome	19 (63.3)	25 (83.3)	
Myocardial infarction	11 (36.7)	5 (16.7)	
**Patients’ level of education**			1.00
Elementary	21 (70)	21 (70)	
High school and over	9 (30)	9 (30)	
**Companions’ level of education**			
Elementary	4 (13.3)	-	-
High school and over	26 (86.7)	-	
**History of hospitalization**			0.79
Yes	18 (60)	19 (63.3)	
No	12 (40)	11 (36.7)	
**Satisfaction of massage**			-
Very much	15 (50)	-	
Highly	14 (46.7)	-	
Moderately	1 (3.3)	-	

**Table 2.** Changes in the Blood Cortisol Level

Cortisol^a^	Intervention, Mean ± SD	Control, Mean ± SD	95% confidence interval of the difference	Test Results
**Before**	323.6 ± 162.6	300.2 ± 198.2	-70.29 , 117.09	P = 0.61
				**t = 0.499**
**After **	268.4 ± 141.1	308.1 ± 191.1	-126.51 , 47.11	P = 0.36
				**t = 0.915**
**P value of Paired t Test**	0.102	0.621	---	---

^a^ Nanomoles

## 5. Discussion

The findings of this research indicated that whole body massage therapy performed by patient's companion decreased the blood cortisol level more than 55 nanomoles. Field et al., have reported that massage leads to a decrease in the level of blood cortisol(19). Besides, Lindgren et al., examined the physiological responses to massage among healthy individuals and reported that salivary cortisol was significantly decreased after massage compared to the pre-massage and one hour after massaging (11). However, there are some conflicting reports. Billhult et al., studied the effect of massage on cellular immunity, endocrine and psychological factors in women with breast cancer and reported no significant difference between the level of salivary cortisol in the control and intervention groups (21). Also, by examining the effect of massage therapy on blood cortisol, Moyer et al. have reported that massage therapy has little effect on the level of blood cortisol (18). Although the changes in the blood cortisol level were not statistically significant in the present study, the observed decrease may be clinically important and signifies the effect of massage therapy. The small sample size may be influenced on the statistical significance of the results. It is also likely that the duration of massage in the present study was not sufficient enough to yield a significant difference among the two groups. Also other variables might have been presented which were out of the researcher's control and were effective on the level of cortisol.

All in all, the findings indicate that massage as an external stimulant may decrease anxiety and cortisol levels. Previous studies have also reported that hypothalamus-hypophysis-adrenal axis is activated during stress and reacts to internal and external stimulants (25). Thus, it could be supposed that massaging may alleviate anxiety which then may lead to a decrease in the blood cortisol level (18).

This research examined the effect of massage therapy on the level of blood cortisol among the male patients hospitalized at the CCU wards. It was indicated that after massage therapy, the level of cortisol had been lowered in the intervention group. Although the resultant decrease was not found to be significant. Regarding the effects of the massage therapy on lowering anxiety and cortisol levels and also its tranquilizing effect, it could be recommended that massage therapy be used in patients admitted in CCU. 

The individual characteristics of researcher and wearing white uniform might have been effective on the level of anxiety and blood cortisol which was an issue out of the researchers' control. Regarding this point that measuring the levels of blood cortisol was performed 15 minutes after the application of massage, it is recommended to conduct further investigations to examine the level of blood cortisol in a longer interval after taking massage so that the durability of blood cortisol reduction is pinpointed.
